# An Investigation of Volatile Flavor Compounds and Lipolysis-Oxidation in Coppa as Affected by the Inoculation of Coagulase-Negative *Staphylococcus* during the Air-Drying Stage

**DOI:** 10.3390/foods13172723

**Published:** 2024-08-28

**Authors:** Jialing Ye, Xuying Zhang, Shuge Yuan, Yuemei Zhang, Jinxuan Cao, Wendi Teng, Ying Wang

**Affiliations:** 1 Key Laboratory of Geriatric Nutrition and Health (Beijing Technology and Business University), Ministry of Education, Beijing 100048, China; yejialing@btbu.edu.cn (J.Y.); zhangyuemei@btbu.edu.cn (Y.Z.); caojinxuan@btbu.edu.cn (J.C.); tengwendi@btbu.edu.cn (W.T.); 2 Beijing Engineering and Technology Research Center of Food Additives, School of Food and Health, Beijing Technology and Business University, Beijing 100048, China

**Keywords:** pork collar butts, *Staphylococcus* fermentation, lipolysis, volatiles

## Abstract

This study aimed to explore the effects of coagulase-negative *Staphylococcus* inoculation on flavor generation and lipolysis-oxidation in Coppa. Acid lipase, neutral lipase, phospholipase, and lipoxygenase (LOX) activities, as well as free fatty acids, volatile compounds, and sensory evaluation, were determined during the fermentation and air-drying processes of Coppa over 40 days. *Staphylococcus carnosus* and *Staphylococcus xylosus* or a combination of both strains were selected for this study, and natural fermentation was treated as a control. The results showed that *Staphylococcus* inoculation significantly enhanced lipase and LOX activities, and mixed strains had a superior effect. Palmitic acid, stearic acid, linoleic acid, and oleic acid were identified as the predominant free fatty acids in Coppa, with the mixed fermentation group exhibiting the highest contents. Acids, aldehydes, alcohols, ketones, esters, and phenols were found for the volatile compounds in Coppa. These findings thus suggested a positive role of *Staphylococcus* inoculation in activating lipolysis-oxidation and contributing to the flavor formation of Coppa during the air-drying stage.

## 1. Introduction

Flavor is a crucial factor in assessing the quality of meat products. It has been recognized that the volatile attributes of meat products are highly associated with the hydrolytic and oxidative processes of lipids occurring during the air-drying process [1]. Lipolysis is initialized by specific lipases, i.e., acid lipase, neutral lipase, and phospholipases, subsequently leading to the release of free fatty acids [2]. The liberated free fatty acids can undergo secondary reactions, including esterification and Maillard reactions, thus enhancing the complexity of flavor through the formation of some aroma-active compounds [3]. Lipid oxidation, catalyzed by reactive oxygen species or enzymatic pathways, results in the degradation of unsaturated fatty acids. This oxidative breakdown yields volatile products, contributing distinct notes to the sensory attributes of meat products [4]. Lipoxygenase (LOX) has been recognized as a pivotal enzyme in lipid oxidation [5], and the activity of LOX has been thoroughly linked to the profile of volatile compounds in dry-cured meat products [6].

Coppa, traditionally prepared from pork collar butts, is one of the most renowned air-dried meat products in Italy, cherished by consumers for its distinctive flavor and rich nutritional profile. The industrialized production of Coppa achieves a high level of maturity, with the fermentation phase considered as a crucial process influencing the qualitative aspects of flavor [7]. Natural fermentation often relies on the indigenous bacteria existing in raw meat to exert their influence. The intricate microbiota during natural fermentation, however, poses difficulties in terms of precise control, resulting in a less stable quality [8]. Consequently, there is a growing interest in exploring superior microbial strains as fermentation agents for application during the production of air-dried meat products [9,10].

Coagulase-negative *Staphylococci*, e.g., *Staphylococcus xylosus* and *Staphylococcus carnosus* have been well utilized as starter cultures during the fermentation of meat products [11]. *Staphylococcus* often exerts its influence on the generation of flavor, primarily through the production of lipases and LOX; these enzymes can further catalyze the oxidation and hydrolysis of lipids, collectively shaping the profile of volatile compounds [12]. *Staphylococcus xylosus* isolated from traditional Chinese Suan yu has demonstrated enhanced lipolytic activity [13]. A correlation of *Staphylococcus* and lipid oxidation has recently been reported in the dry-cured duck. When used in combination, *Staphylococcus xylosus* and *Staphylococcus carnosus* can have a synergistic effect, enhancing the overall flavor complexity of fermented meats [14]. *Staphylococcus xylosus* contributes to strong lipolytic activity and flavor compound formation, while *Staphylococcus carnosus* supports moderate enzymatic activity. The specifics of this process are, however, primarily dependent on factors such as strain, fermentation conditions, and substrate composition. The choice of bacterial strain, particularly within species like *Staphylococcus xylosus* and *Staphylococcus carnosus*, is a critical determinant of the fermentation outcome. The specific conditions under which fermentation occurs, including temperature, humidity, and time, significantly influence the activity of the microbial cultures and the overall fermentation process. Also, the composition of the meat substrate, including its fat content, protein levels, and overall nutrient profile, directly influences the fermentation process and the final product characteristics.

This study aimed to investigate the impact of *Staphylococcus* inoculation on the flavor quality and lipolysis-oxidation of Coppa by introducing microbial strains (*Staphylococcus carnosus*, *Staphylococcus xylosus*, and a combination of both) during the air-drying process. The headspace solid-phase microextraction gas chromatography–mass spectrometry (HS-SPME) was used for the enrichment of volatile flavor compounds, coupled with GC-MS to separate and identify volatile flavor compounds. Additionally, the activities of lipase, phospholipase, and lipoxygenase, as well as free fatty acid contents, were determined to clarify the role of lipolysis-oxidation in controlling the flavor profile of Coppa. This study may contribute to enhancing our understanding of the origin of lipid-derived flavors in dry-cured meat products.

## 2. Materials and Methods

### 2.1. Processing of Fermented Coppa

#### 2.1.1. Preparation of *Staphylococcus* Strains

*Staphylococcus carnosus* (strain number 337536) and *Staphylococcus xylosus* (strain number 337469) were purchased from Beina Biotech Co., Ltd, Beijing, China. Tryptic Soy Broth (TSB) was used to solubilize freeze-dried *Staphylococcus* strains. Following culturing at 37 °C for 24 h, a sterile inoculation loop was used to transfer the liquid in the tube, which was then streaked repeatedly on tryptic soy agar plates to activate the bacteria. Gram staining and further microscopic examination were performed to check the target colony. For activation of *Staphylococcus xylosus* strains, nutrient broth and nutrient agar were prepared for solubilization and further repeated plate streaking.

#### 2.1.2. Fermentation and Processing of Coppa

Deboned pork collar butts at 48 h postmortem were purchased from the Jiaxing meat wholesale market. The preparation of the pork collar butts was informed by established methodologies found in a recent study [15]. After trimming of skin and visible connective tissue, a proportion of pork collar butts weighing 1.5 ± 0.2 kg was collected. Mixed seasonings of edible salt (35 g/kg), white pepper powder (2 g/kg), clove powder (0.5 g/kg), kudzu root powder (0.5 g/kg), nutmeg (0.5 g/kg), sugar (3 g/kg), sodium erythorbate (0.5 g/kg), and sodium nitrite (0.1 g/kg) were applied uniformly to meat surface. The curing process was performed at 2 °C and 80~85% relative humidity for 20 days. After curing, the meat surface was immersed in clean water and then hung in a cooling box at 2 °C for 1 day for dehumidification. The incubated strains were cultured until reaching a content of 10^7^ CFU/mL, and each piece of meat within each treatment was subsequently sprayed individually with a solution equivalent to 1% of its weight. The ratio of *Staphylococcus carnosus* to *Staphylococcus xylosus* in the mixed bacterial solution was set as 1:1. Four groups were assigned in the further procedure: (1) natural fermentation without bacterial inoculation as a control (NA), (2) inoculation only with *Staphylococcus carnosus* (RS), (3) inoculation only with *Staphylococcus xylosus* (MS), and (4) inoculation with a mixed bacterial solution of *Staphylococcus carnosus* and *Staphylococcus xylosus* (HS). The natural fermentation and inoculation fermentation were both conducted at 30 °C and 80% relative humidity for 24 h, followed by air-drying for 40 d at 18 °C and 75% relative humidity. In this experiment, precise control and monitoring of relative humidity were achieved using a fermentation chamber equipped with a Programmable Logic Controller (PLC). The PLC system allowed for accurate regulation of both temperature and humidity, creating a controlled microenvironment suitable for the specific drying and fermentation processes. At the end of fermentation, the weight loss rate of the product reached approximately 35%, and the water activity decreased to around 0.83. Six samples from each group were taken at 0 days (end of curing), 10 days, 20 days, 30 days, and 40 days of air-drying, respectively. All samples were cut into small pieces, wrapped in aluminum foil, labeled, and stored at −20 °C for further experiments.

### 2.2. Sensory Evaluation

The sensory evaluation, including basic meat aroma, characteristic meat aroma, fermented meat aroma, spice aroma, and overall perceived aroma, was conducted, with intensity scores ranging from 1 to 10 for each attribute (1–3: weak; 4–6: moderate; 7–10: strong). The pieces were judged by a trained panel (five men and five women, aged 24–32 years) with expertise in evaluating fermented meat products, and written consent was signed by all individual participants before the trial. During the evaluation process, Coppa samples from each group were sliced into 1 mm thick slices and assessed by each panelist individually under room temperature and natural light conditions.

### 2.3. Lipase Activity Determination

#### 2.3.1. Extraction of Crude Lipases

The crude lipase extraction was conducted as in reference [2], with slight modifications. Approximately 200 mg of finely ground sample was added to 3 mL of 50 mM phosphate buffer (5 mM ethylene glycol-bis (2-aminoethyl ether)-N, N, N’, N’-tetraacetic acid, pH 7.5) and homogenized at 10,000 rpm for 30 s followed by centrifuging at 4 °C and 8000 rpm for 20 min. The supernatant was filtered to remove the upper layer of fat and used as crude lipase solution for further analysis. The protein content in the supernatant was determined using the bicinchoninic acid method.

#### 2.3.2. Determination of Neutral Lipase Activity

Neutral lipase activity was measured following the reference [16] with slight modifications. Crude enzyme extracts (10 μL) were mixed with a 280 μL solution of 0.22 M Tris/HCl buffer (pH 7.5) containing 0.05% (*w*/*v*) Triton X-100, and 10 μL of 1.0 mM 4-methylumbelliferyl oleate as the substrate was then added into the above mixture. After incubation at 37 °C for 30 min, the mixture was immediately cooled in an ice bath, and the fluorescence intensity was immediately measured at an excitation wavelength of 350 nm and an emission wavelength of 445 nm. The enzyme extraction buffer was used as a blank control instead of the enzyme extraction solution.

#### 2.3.3. Determination of Acid Lipase Activity

Acid lipase activity was measured following the reference [16]. Crude enzyme extracts (10 μL) were diluted with a 280 μL solution of 0.1 M disodium phosphate/0.05 M citric acid buffer (pH 5.0) containing 0.05% (*w*/*v*) Triton X-100 and 0.8 mg/mL bovine serum albumin (BSA), and subsequently, 10 μL of 1.0 mM 4-methylumbelliferyl oleate as the substrate were added. After incubation at 37 °C for 30 min, the reaction was terminated with 10 μL of 1M HCL, and the fluorescence intensity was measured at an excitation wavelength of 350 nm and an emission wavelength of 445 nm. The enzyme extraction buffer was used as blank control.

#### 2.3.4. Determination of Phospholipase Activity

Phospholipase activity was determined following the reference [16] with slight modifications. Crude enzyme extracts (10 μL) were diluted with a 280 μL solution of 0.1 M disodium phosphate/0.05 M citric acid buffer (pH 5.0) containing 0.05% (*w*/*v*) Triton X-100, 0.8 mg/mL BSA, and 150 mM sodium fluoride and 10 μL of 1.0 mM 4-methylumbelliferyl oleate as the substrate were added. After incubation at 37 °C for 30 min, the reaction was terminated with 10 μL of 1M HCl, and the fluorescence intensity was measured at an excitation wavelength of 350 nm and an emission wavelength of 445 nm.

The activities of lipase and phospholipase were calculated using a standard curve. The amount of enzyme hydrolyzing 1 μmol substrate per hour at 37 °C was defined as one unit of activity (U). The activities of neutral lipase, acid lipase and phospholipase were expressed as U/g enzyme protein.

### 2.4. Determination of LOX Activity

The extraction and activity measurement of LOX was conducted following the reference [17] with slight modifications. Approximately 200 mg of finely ground Coppa was added to 3 mL of 50 mM phosphate buffer (containing 1 mM dithiothreitol, 1 mM ethylenediaminetetraacetic acid, pH 7.5) and homogenized at 10,000 rpm in an ice bath for three cycles of 10 s each. The homogenate was then centrifuged at 4 °C and 8000 rpm for 20 min. The supernatant was collected and filtered as crude LOX solution for activity analysis. The protein content in the supernatant was determined using the bicinchoninic acid method.

Linoleic acid was used to prepare the substrate solution. Approximately 140 mg of linoleic acid was solubilized with 5 mL of deoxygenated double-distilled water containing 180 μL of Tween 20 and then adjusted to pH 9.0 by adding 2 M NaOH. The mixture was diluted to 50 mL with distilled water and kept under nitrogen atmosphere. Enzyme extraction solution (20 μL) was added into a mixture of 380 μL of 50 mM citric acid buffer (pH 5.5) and 40 μL of linoleic acid substrate solution. After incubation at 37 °C for 1 min, the absorbance of the mixture was measured at 234 nm before and after incubation. A blank control was prepared using 400 μL of citric acid buffer and 40 μL of substrate solution instead of enzyme extraction solution. Under conditions of 37 °C, the increase in absorbance at 234 nm per minute was defined as one unit of LOX activity (U), and LOX activity was expressed as U/g enzyme protein.

### 2.5. Determination of Free Fatty Acids

#### 2.5.1. Extraction of Intramuscular Lipids

Extraction of intramuscular lipids was performed following a previous reference [2] with slight modifications. Minced sample (around 3 g) was homogenized with a chloroform and methanol mixture at a ratio of 2:1 to a final volume of 45 mL at 3000 rpm for 10 s. After standing for 2 h, the mixtures were filtered, and the filtrate was then washed with physiological saline at 0.2 times the volume. The washed solution was centrifuged at 4500 rpm for 15 min, and the lower layer was collected in a rotary evaporator and vacuum-dried. The dried lipid was stored at −20 °C for further analysis.

#### 2.5.2. Separation and Determination of Free Fatty Acids

The dried lipids (20 mg) were dissolved in 1 mL of chloroform. Then, 0.5 mL of the solution was transferred to an aminopropylsilica minicolumn (100 mg, Varian, Beijing, China) and activated with 1 mL of chloroform. Neutral lipids were first eluted out of minicolumn using 2 mL of 2-propanol/chloroform (1:2, *v*/*v*), and consequently, free fatty acids were eluted with 3 mL of ether-acetic acid (2%, *w*/*w*) and dried under nitrogen for further determination.

Following the method [2], dried free fatty acids were mixed with 2 mL of boron trifluoride–methanol (mass fraction 14%), and the mixture was heated at 60 °C for 30 min for methylation, followed by adding 2,2-dimethoxypropane to remove moisture. Heptadecanoic acid was chosen as an internal standard. After cooling, 1 mL of hexane and 1 mL of distilled water were added to the mixture, shaken, and left to stand for 1 h. The upper layer solution was dried under nitrogen and dissolved in 0.5 mL of hexane for analysis. Quantification of free fatty acids was performed using an external standard method. Gas chromatography was used with a capillary column (50 m × 0.25 mm × 0.20 μm) to analyze methylated fatty acid samples (1.5 μL). The temperature of injection port and detector was set at 280 °C, and the oven temperature was ramped from 160 °C to 220 °C at a rate of 6 °C/min and then held at 220 °C for 30 min. The carrier gas pressure was maintained at 80 kPa with a split ratio of 1:40.

### 2.6. Determination and Analysis of Volatile Flavor Compounds

Volatile flavor compounds were determined using the HS-SPME GC-MS method [2] with slight modifications. Minced Coppa samples (around 4 g) were placed in a headspace vial (CNW Technologies, Dusseldorf, Germany), and 10 μL of 2-octanol was added as an internal standard, followed by sealing with PTFE/silicone septum. After equilibration for 25 min at 45 °C, volatiles in the headspace were extracted by a SPME device (Supelco Inc., Bellefonte, PA, USA) equipped with a 75 μm carboxen/polydimethylsiloxane (CAR/PDMS) fiber. It was preconditioned at 220 °C for 2 h and then extracted for 40 min at 45 °C. The fiber was inserted into the injector aimed at desorbing for 4 min at 220 °C set in the splitless mode.

The GC-MS QP2010 plus (Shimadzu, Kyoto, Japan) was used for analyzing the level of volatile compounds, and helium was set as the carrier gas. After desorbing, the oven temperature ramp was held at 40 °C for 4 min and heated to 245 °C at 5 °C/min for 15 min. Ion source temperature was set as 230 °C, and the mass range was set from *m*/*z* 20 to 400. Identification was based on comparing the retention time of standard compounds (Sigma-Aldrich, Steinheim, Germany) and mass spectra stored in the NIST and WILEY7.0 databases (Gaithersburg, MD, USA).

### 2.7. Data Analysis

Statistical analysis was conducted using one-way analysis of variance (ANOVA) with Duncan’s multiple comparison method in IBM SPSS Statistics 26 software. Triplicates were performed for the above analysis. Data were shown as means ± standard deviations. The significance was set at *p* < 0.05 between group means. Hierarchical cluster analysis were operated by OriginPro 2021 aimed to analyze the relationship among the observed data between the differential fermentation groups.

## 3. Results

### 3.1. Sensory Changes

**Figure 1:**
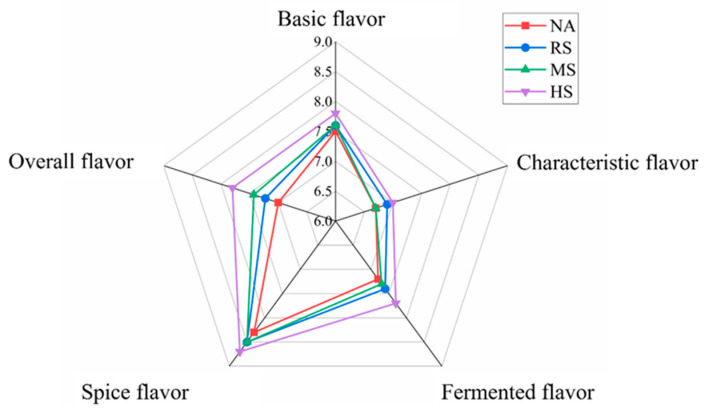
Aroma sensory evaluation of Coppa samples among the four groups (Natural fermentation group, NA; *Staphylococcus carnosus* group, RS; *Staphylococcus xylosus* group, MS; Mixed *Staphylococcus* strains group, HS).

Sensory analysis for Coppa flavor under different fermentation conditions is shown in Figure 1. In terms of overall flavor and fermented flavor scores, the HS group outperformed the other three groups significantly (*p* < 0.05). The ranking of the groups based on these scores is HS > MS > RS > NA, indicating that the HS fermentation condition produced the most favorable flavor profile, while the NA group had the lowest scores. The scores for basic meat flavor, characteristic meat flavor, and spice flavor were relatively consistent across all four groups, suggesting that these particular flavor attributes were less influenced by the variations in fermentation conditions. However, it is noteworthy that the naturally fermented group scored lower in these categories (*p* < 0.05), which could be attributed to the uncontrolled fermentation environment, leading to a less consistent development of these flavors.

### 3.2. Lipase and LOX Activities

**Figure 2:**
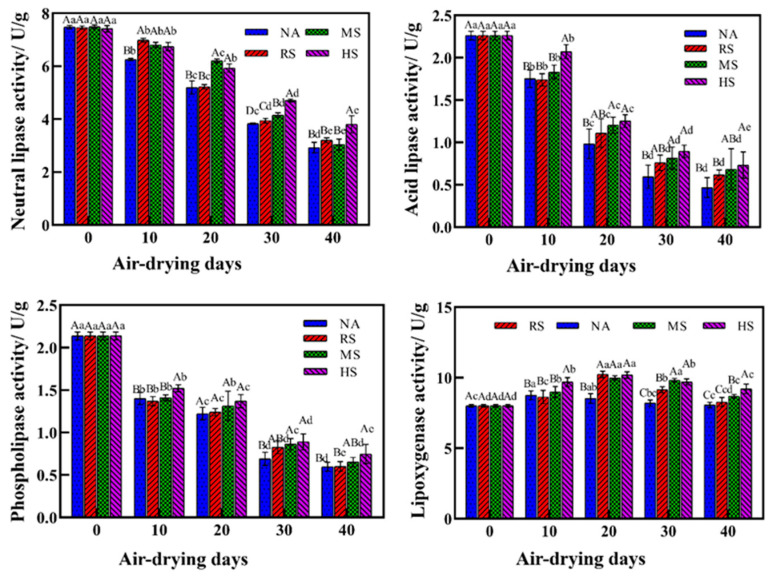
Changes in neutral lipase, acid lipase, phospholipase, and LOX activities in Coppa with or without inoculation of *Staphylococcus* strains during the air-drying process. Means ± standard errors are shown. Different lowercase letters (a–e) represent significant variations in the different air-drying days within the same group (*p* < 0.05), and different uppercase letters (A–D) indicate significant inter-group differences at the same air-drying stage (*p* < 0.05).

As shown in Figure 2, the activities of three lipases in all four treatment groups significantly decreased during the air-drying process (*p* < 0.05). The HS group showed higher values (*p* < 0.05) of lipase activities as compared to the NA group throughout the air-drying process. LOX activities in the four groups showed an increasing trend during the initial 20 days and then decreased slightly in the subsequent 20 days. The HS group also showed higher LOX activities than the NA group throughout the air-drying process (*p* < 0.05), indicating a potential role of mixed *Staphylococcus* in promoting enzymatic actions catalyzed by the LOX.

### 3.3. Free Fatty Acids

Four groups showed similar fatty acid compositions following the fermentation and air-drying process (Table 1), with 11 substances detected. Saturated fatty acids were the most abundant among the four groups, followed by monounsaturated fatty acids and polyunsaturated fatty acids. Among free fatty acids, palmitic acid (C16:0), stearic acid (C18:0), linoleic acid (C18:2), and oleic acid (C18:1) exhibited relatively higher contents and can be regarded as the main free fatty acids of Coppa.

### 3.4. Volatile Flavor Compounds

The composition of volatile flavor compounds in the four groups was relatively complex, as shown in Table 2. Among volatile compounds, acids exhibited the highest level, followed by aldehydes, alcohols, ketones, esters, and phenols, with alcohols and esters having the highest diversity. Alcohol compounds contributed significantly to the aroma profile, including olefinic alcohols, straight-chain alcohols, and branched-chain alcohols. The levels of 2-hexanol, isopropyl alcohol, ethanol, 3-methyl-3-buten-1-ol, and terpinen-4-ol were significantly higher in the fermentation groups compared to the NA group. The 2,3-butanediol provides a bitter taste, and its level in the NA group was significantly higher than in the other fermentation groups. Aldehydes also exert an influence on the aroma of processed meat products [9]. The levels of benzeneacetaldehyde and hexanal in the fermentation groups were significantly higher than that in the NA group. There were significant differences in the levels of six acidic compounds between the NA and fermentation inoculation groups, namely acetic acid, n-decanoic acid, hexanoic acid, isobutyric acid, butanoic acid, and propanoic acid. *Staphylococcus* fermentation, particularly for mixed strains, induced a significant decrease in the 3-hydroxy-2-butanone when compared with the NA group. However, butanone and 6-methyl-5-hepten-2-one were significantly higher in the HS group. The level of ethyl isovalerate was the highest (*p* < 0.05) in the four groups, imparting an aroma reminiscent of apples and mulberries. The ethyl propionate, ethyl acetate, ethyl 2-methylpropanoate, ethyl hexanoate, triethyl phosphate, ethyl octanoate, and ethyl butanoate were significantly higher in the inoculated fermentation groups than in the NA group. Most of the above esters exhibit fruity aromas and may be considered significant contributors to the flavor of Coppa during fermentation. Increased eugenol and p-Cresol were also found in the inoculation groups as compared to the NA group.

### 3.5. Hierarchical Cluster Analysis

**Figure 3:**
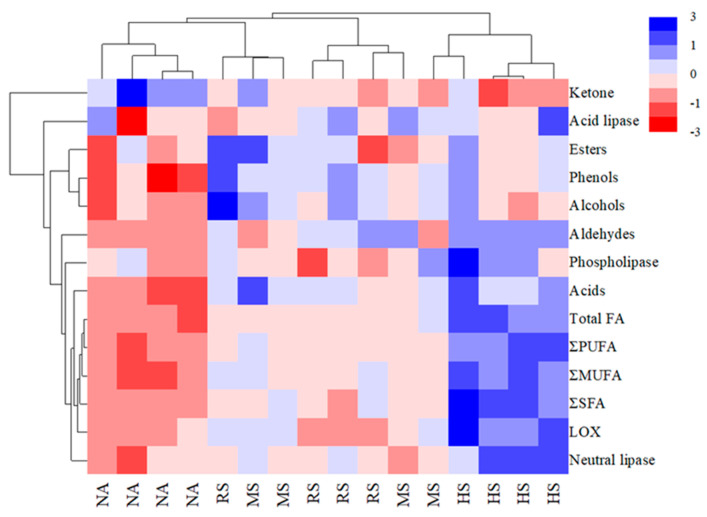
Heat map of hierarchical cluster analysis between the four differential groups. Total saturated fatty acid (ΣSFA); total monounsaturated fatty acids (ΣMUFA); total polyunsaturated fatty acids (ΣPUFA); total free fatty acids (TOTAL FA).

Hierarchical cluster analysis (HCA) was used to identify patterns and similar group samples based on their color attributes and other measured variables. The legend in the figure categorizes samples into distinct groups, with each group represented by a unique color for clarity. The color values ranging from −3 to +3 in the hierarchical cluster analysis are used to visually encode the range of attribute values, with red colors indicating lower attribute values and blue colors indicating higher attribute values. As shown in Figure 3, significant differences were found between the NA and the *Staphylococcus* groups in terms of the color attribute, and the samples from the NA group and the HS group were divided into two different clusters according to the observed determinations. The HS group showed up-regulated lipase and LOX activities, relative contents of free fatty acids, and varied differential volatile compounds, as compared to the NA group. These biochemical differences contributed to the observed variation in color attributes and were clearly shown through the clustering results.

## 4. Discussion

This is the first study reporting an effect of *Staphylococcus* fermentation on the volatile flavor of Coppa, particularly in relation to the lipolysis-oxidation for achieving artificial control during fermentation and the air-drying process. The *Staphylococcus* fermentation accumulated numerous volatiles, particularly in the case of mixed cultures (Table 2). Odor sensory evaluation indicated that the mixed inoculation group received the highest scores (Figure 1). This can be directly attributed to increased unsaturated fatty acids found in Coppa (Table 1). Accordingly, the observed changes in lipase and lipoxygenase activities (Figure 2) would potentially facilitate the breakdown of lipids and further formation of free fatty acids during the fermentation and drying process. Our results thus suggest a role of *Staphylococcus* fermentation in promoting the formation of lipid-derived flavor in Coppa. The sensory evaluation of flavor was conducted with a specific panel of participants under controlled conditions, offering more robust insights into how *Staphylococcus* fermentation influences flavor perception in different demographic groups. *Staphylococcus* inoculation firstly activated lipases and subsequently promoted the hydrolysis of lipids, resulting in the release of unsaturated fatty acids, which would serve as substrates for LOX to facilitate the enzymatic oxidation reactions, thus contributing to the enhanced flavor profile of Coppa. The combination of different *Staphylococcus* strains exhibited a synergistic effect, potentially offering a broader substrate utilization spectrum. This would allow them to access and metabolize a wider variety of lipid molecules, as evidenced by the elevated levels of free fatty acids observed in the mixed *Staphylococcus* inoculation group compared to the single inoculation group. Interaction between strains may influence the expression of lipolytic enzymes. By leveraging the unique properties of each strain, the synergistic effect results in a more effective breakdown of lipids and a richer array of flavor compounds, surpassing the outcomes achievable by single strains alone.

*Staphylococcus* species, particularly *Staphylococcus xylosus* and *Staphylococcus carnosus*, are known to play a significant role in the development of lipid-derived flavors in fermented products. This role can be attributed to their ability to produce specific enzymes that interact with lipids to produce flavor compounds. Lipases are the primary enzymes produced by *Staphylococcus* species that initiate the breakdown of lipids into free fatty acids and glycerol [2]. The free fatty acids released are crucial precursors for flavor development. The lipase activities in the HS group were significantly higher than that in the NA group throughout the air-drying storage, which could be highly attributed to the inoculation of mixed *Staphylococcus* secreting more lipases [18]. Increased lipase activity would promote more efficient hydrolysis of triglycerides into free fatty acids and other flavor precursors, resulting in a richer, more complex and appealing flavor profile [3]. Some metabolites produced by *Staphylococcus* could facilitate more efficient catalysis by the lipases [19]. The specificity of LOX for free fatty acids and the regiospecificity of oxygen insertion contribute to the diversity of lipid mediators produced [20]. Several studies have reported an important role of LOX activity in the formation of special flavors in meat products [5,21]. The LOX activity exhibited an initial increase followed by a subsequent decrease in all groups during air-drying storage (Figure 2), consistent with the findings in previous studies of dry-cured meat products [6]. Alterations in water activity or exposure to oxygen, along with air-drying and inoculation fermentation processes, might be responsible for the expression and activity of LOX [22]. Samples inoculated with *Staphylococcus* strains showed higher values (*p* < 0.05) of LOX activity, especially in the mid-stage of the air-drying storage (Figure 2). This was highly related to the observed increase in polyunsaturated fatty acids due to *Staphylococcus* fermentation (Table 2). Growth and metabolic activities of *Staphylococcus* interacted with decreased water activity during the air-drying process would generate various metabolic byproducts, some of which could potentially influence the activity of LOX. The study employs controlled experimental controls, including the use of mixed cultures and standardized conditions for fermentation and drying. This approach ensures that the observed effects are attributable to the fermentation process and provides a robust framework for future research in this area.

Free fatty acids serve as precursors and derivatives for volatiles, exerting a significant impact on the flavor of air-dried meat products. The SFA component was predominantly characterized by the prevalence of palmitic acid and stearic acid, and these specific SFAs have been well reported to constitute the primary saturated fatty acids found in various cured meat products [23]. The total contents of free fatty acids in this study followed the order of HS group > MS group > RS group > NA group (Table 1). Increased free fatty acid contents have been reported in Chinese dry fermented sausages with *Staphylococcus xylosus* inoculation [24] and in dry fermented mutton sausages with *Staphylococcus carnosus* fermentation [25]. The highest contents of SFA, MUFA, PUFA, and total fatty acids both appeared in the HS group, underscoring the enzyme-mediated transformations within the lipid profile. Thus, herein, we ascribed the increased fatty acid contents found in the mixed *Staphylococcus* fermentation group to an accelerated reaction of lipolysis. The integration of these processes with sensory evaluation offers a deeper understanding of how lipolytic and oxidative reactions shape the final flavor profile.

The effect of the fermentation mediated by *Staphylococcus*, individually and in combination, on the enrichment of volatiles in Coppa is shown in Table 2. Alcohols exhibit a close correlation with the process of lipid oxidation [26]. Among alcohol compounds, 2-hexanol possesses a sweet mushroom aroma, and isopropyl alcohol exhibits a refreshing fragrance, adding a clean and crisp note. Characterized by an aromatic scent, 3-methyl-3-buten-1-ol enriches the flavor profile, adding a distinctive fragrant note that complements other aromatic compounds. Terpinen-4-ol exhibits a peppery aroma, which is likely derived from the white pepper added in the seasoning spices during curing. These characteristic alcohol compounds were found to increase following the *Staphylococcus* fermentation. The 2,3-butanediol, known for its bitter flavor, exhibited a significant decrease in the fermentation groups compared to the NA group. This was in agreement with the findings of [27] at the end of the fermentation of Harbin dry sausages. Aldehyde compounds exhibit lower flavor threshold values, suggesting an important role in influencing the aroma of air-dried meat products [6,23]. Benzeneacetaldehyde is often described as having a pleasant and somewhat fruity scent. *Staphylococcus* fermentation significantly increased the level of benzeneacetaldehyde as compared to the NA group. It has been reported that short-chain acids (C < 6) resulting from lipid oxidation exerted a more pronounced influence on the aroma of fermented meat products [28]. Acetic acid was the most abundant acidic compound in all groups, which was in agreement with the findings [29,30] for fermented sausages. Acids are frequently not regarded as predominant contributors to aroma, given their relatively high odor thresholds. However, acidic compounds in conjunction with alcohols have been well-documented to be significant precursors for ester formation [31]. Esters often impart fruity aroma and sweetness and, thus, play an important role in masking undesirable odors [32]. Ethyl isovalerate, characterized by a fragrance reminiscent of apple and mulberry, was observed as the most abundant ester compound in the air-dried Coppa. The characteristic ester compounds in this study have been reported earlier to contribute to the aroma profile of dry fermented sausage [28,30]. The levels of esters such as ethyl propionate, ethyl acetate, ethyl 2-methylpropanoate, and ethyl butanoate were significantly higher in the inoculated fermentation groups compared to the NA group (Table 2). Ethyl propionate contributes a sweet, fruity aroma. Ethyl acetate adds a light, fruity note with a hint of pear. Ethyl 2-methylpropanoate imparts a fruity, slightly tropical aroma. Ethyl butanoate provides a pineapple-like scent, further enhancing the fruity and sweet notes in Coppa. These enhanced esters could, thus, enrich the complexity of the flavor profile in the *Staphylococcus* fermentation samples. Ketone compounds represented significant constituents in Coppa, with many of them characterized by creamy and fruity notes. The 3-hydroxy-2-butanone, commonly known as acetoin, is characterized by a flavor profile that imparts a buttery and overripe fruit aroma. Wen et al. observed a similar decrease in 3-hydroxy-2-butanone in dry fermented sausages inoculated with yeast strains [30]. *Staphylococcus* fermentation in this study significantly increased the levels of methyl ketones, i.e., butanone and 6-methyl-5-hepten-2-one. It has been reported that methyl ketones, as byproducts of lipid oxidation, could be recognized as key precursors contributing to the development of characteristic fatty aroma [33]. Methyl ketones are often regarded as key precursors that enhance the savory and fatty notes, providing depth to the overall flavor. By demonstrating how *Staphylococcus* fermentation can be used to manipulate and enhance flavor profiles in Coppa, this study provides practical implications for the meat processing industry.

## 5. Conclusions

The inoculation of *Staphylococcus carnosus*, *Staphylococcus xylosus,* or a combination of both strains as fermentation agents in Coppa significantly enhanced lipase activities, including acid lipase, neutral lipase and phospholipase along with LOX, throughout the air-drying process. This enhancement contributed to the breakdown of lipids, facilitating the observed release of free fatty acids and the generation of volatile compounds. Fermentation with mixed *Staphylococcus* strains significantly increased the levels of volatile compounds, particularly for some acidic and ester compounds. Sensory evaluation results further demonstrated that the mixed fermentation group achieved the highest scores, underscoring a positive impact of combined *Staphylococcus* strains on the overall sensory attributes. Hence, *Staphylococcus carnosus* and *Staphylococcus xylosus* can be considered superior microbial strains for the production of air-dried Coppa products. Further studies can examine the interactions between these strains and other micro-organisms (e.g., bacteria and yeasts) to provide a more comprehensive understanding of their roles in the fermentation process.

## Figures and Tables

**Table 1:** Effect of *Staphylococcus* inoculation on free fatty acids composition (g/kg lipid) of Coppa.

Free Fatty Acids	NA	RS	MS	HS
C16:0	15.58 ± 0.98 ^c^	17.86 ± 1.13 ^b^	18.85 ± 1.07 ^b^	25.99 ± 1.23 ^a^
C16:1	0.10 ± 0.01 ^d^	0.26 ± 0.01 ^b^	0.16 ± 0.01 ^c^	0.33 ± 0.01 ^a^
C16:1 T	0.19 ± 0.01 ^c^	0.39 ± 0.02 ^b^	0.39 ± 0.04 ^b^	0.56 ± 0.07 ^a^
C18:0	7.30 ± 0.53 ^c^	8.47 ± 0.66 ^b^	8.19 ± 0.56 ^bc^	11.64 ± 0.82 ^a^
C18:2	8.74 ± 1.62 ^d^	12.68 ± 1.08 ^c^	15.48 ± 0.85 ^b^	21.35 ± 1.03 ^a^
C18:1	9.67 ± 0.71 ^c^	16.39 ± 0.83 ^b^	15.63 ± 0.67 ^b^	21.69 ± 0.94 ^a^
C18:3	0.26 ± 0.02 ^c^	0.47 ± 0.06 ^b^	0.39 ± 0.11 ^b^	0.99 ± 0.11 ^a^
C20:2	0.14 ± 0.01 ^d^	0.23 ± 0.01 ^b^	0.18 ± 0.01 ^c^	0.42 ± 0.01 ^a^
C20:3	0.06 ± 0.01 ^b^	0.09 ± 0.01 ^ab^	0.08 ± 0.01 ^b^	0.11 ± 0.03 ^a^
C20:4	2.20 ± 0.12 ^b^	2.76 ± 0.31 ^ab^	2.32 ± 0.43 ^b^	3.36 ± 0.62 ^a^
C22:4	0.06 ± 0.01 ^d^	0.29 ± 0.01 ^b^	0.25 ± 0.01 ^c^	0.45 ± 0.01 ^a^
ΣSFA	22.88 ± 1.57 ^c^	26.33 ± 2.07 ^bc^	27.04 ± 2.18 ^b^	37.63 ± 3.27 ^a^
ΣMUFA	9.96 ± 1.07 ^c^	17.04 ± 1.54 ^b^	16.18 ± 1.38 ^b^	22.58 ± 1.76 ^a^
ΣPUFA	11.96 ± 1.54 ^c^	16.52 ± 1.36 ^b^	18.70 ± 1.35 ^b^	26.68 ± 1.93 ^a^
TOTAL	44.80 ± 2.87 ^c^	59.89 ± 3.17 ^b^	61.92 ± 3.08 ^b^	86.89 ± 3.23 ^a^

^a–d^ Same letters in the row indicate no significant difference (*p* > 0.05). Natural fermentation group (NA); *Staphylococcus carnosus* group (RS); *Staphylococcus xylosus* group (MS); Mixed *Staphylococcus* strains group (HS); total saturated fatty acid (ΣSFA), total monounsaturated fatty acids (ΣMUFA), and total polyunsaturated fatty acids (ΣPUFA).

**Table 2:** Effect of *Staphylococcus* inoculation on volatile compounds identified and semi-quantified in the Coppa samples.

Volatile Compounds	RT	NA	RS	MS	HS
Alcohols					
1-Pentene-3-ol	8.931	0.062 ± 0.008 ^a^	0.103 ± 0.019 ^a^	0.121 ± 0.022 ^a^	0.158 ± 0.039 ^a^
1-Octen-3-ol	17.044	0.143 ± 0.026 ^a^	0.260 ± 0.060 ^a^	0.171 ± 0.019 ^a^	0.181 ± 0.007 ^a^
1-Pentanol	11.570	0.073 ± 0.011 ^a^	0.102 ± 0.016 ^a^	0.092 ± 0.003 ^a^	0.097 ± 0.013 ^a^
endo-Borneol	23.004	0.008 ± 0.002 ^a^	0.016 ± 0.005 ^a^	0.011 ± 0.002 ^a^	0.009 ± 0.002 ^a^
Methanol	2.653	0.094 ± 0.012 ^a^	0.123 ± 0.012 ^a^	0.149 ± 0.030 ^a^	0.107 ± 0.008 ^a^
3-Pentanol	13.865	0.122 ± 0.062 ^a^	0.173 ± 0.073 ^a^	0.197 ± 0.085 ^a^	0.164 ± 0.083 ^a^
Octanol	19.767	0.029 ± 0.006 ^a^	0.03 ± 0.003 ^a^	0.025 ± 0.001 ^a^	0.023 ± 0.003 ^a^
Benzyl alcohol	26.669	0.015 ± 0.002 ^a^	0.028 ± 0.005 ^a^	0.023 ± 0.001 ^a^	0.022 ± 0.002 ^a^
1-Heptanol	17.193	0.014 ± 0.002 ^a^	0.021 ± 0.001 ^a^	0.021 ± 0.001 ^a^	0.016 ± 0.001 ^a^
2-Hexanol	9.205	0.008 ± 0.001 ^b^	0.011 ± 0.003 ^b^	0.051 ± 0.008 ^a^	0.015 ± 0.002 ^b^
Phenylethyl alcohol	27.402	0.519 ± 0.006 ^a^	0.896 ± 0.255 ^a^	0.829 ± 0.086 ^a^	0.747 ± 0.154 ^a^
Isopropyl alcohol	3.096	0.009 ± 0.002 ^b^	0.025 ± 0.005 ^a^	0.033 ± 0.008 ^a^	0.023 ± 0.001 ^a^
Ethanol	3.178	0.146 ± 0.024 ^a^	0.246 ± 0.010 ^b^	0.263 ± 0.049 ^b^	0.232 ± 0.019 ^b^
2,3-Butanediol	20.247	0.134 ± 0.033 ^a^	0.008 ± 0.003 ^b^	0.016 ± 0.000 ^b^	0.015 ± 0.008 ^b^
3-Methyl-3-buten-1-ol	11.435	0.018 ± 0.004 ^b^	0.026 ± 0.002 ^b^	0.039 ± 0.002 ^a^	0.037 ± 0.002 ^a^
Terpinen-4-ol	20.708	0.530 ± 0.102 ^c^	1.234 ± 0.266 ^a^	0.952 ± 0.041 ^ab^	0.895 ± 0.123 ^ab^
(E)-2-Octen-1-ol	21.104	0.005 ± 0.001 ^a^	0.005 ± 0.001 ^a^	0.004 ± 0.000 ^a^	0.007 ± 0.002 ^a^
Cedrol	31.474	0.001 ± 0.000 ^a^	0.003 ± 0.001 ^a^	0.003 ± 0.000 ^a^	0.003 ± 0.001 ^a^
Propyl alcohol	5.386	0.229 ± 0.028 ^a^	0.258 ± 0.074 ^a^	0.255 ± 0.018 ^a^	0.074 ± 0.014 ^b^
Isopentanol	10.336	0.420 ± 0.114 ^a^	0.504 ± 0.103 ^a^	0.417 ± 0.038 ^a^	0.318 ± 0.046 ^a^
Aldehydes					
Propanal	1.874	0.016 ± 0.004 ^a^	0.013 ± 0.005 ^a^	0.016 ± 0.003 ^a^	0.018 ± 0.006 ^a^
Nonanal	15.386	0.126 ± 0.051 ^a^	0.112 ± 0.013 ^a^	0.090 ± 0.013 ^a^	0.141 ± 0.037 ^a^
Pentanal	3.860	0.056 ± 0.002 ^a^	0.085 ± 0.009 ^a^	0.095 ± 0.013 ^a^	0.095 ± 0.016 ^a^
Benzeneacetaldehyde	21.383	0.219 ± 0.059 ^b^	2.856 ± 0.300 ^a^	1.155 ± 0.708 ^a^	3.097 ± 0.327 ^a^
Piperonal	33.319	0.012 ± 0.007 ^a^	0.010 ± 0.001 ^a^	0.008 ± 0.001 ^a^	0.012 ± 0.002 ^a^
β-Cyclocitral	16.725	0.003 ± 0.000 ^a^	0.005 ± 0.001 ^a^	0.004 ± 0.001 ^a^	0.003 ± 0.000 ^a^
Benzaldehyde	18.519	0.187 ± 0.068 ^a^	0.336 ± 0.035 ^ab^	0.242 ± 0.093 ^a^	0.427 ± 0.066 ^ab^
Butanal	2.4350	0.014 ± 0.007 ^a^	0.012 ± 0.004 ^a^	0.006 ± 0.001 ^a^	0.009 ± 0.002 ^a^
1-Piperidinecarboxaldehyde	24.386	0.003 ± 0.000 ^a^	0.005 ± 0.000 ^a^	0.004 ± 0.000 ^a^	0.004 ± 0.000 ^a^
Octanal	12.457	0.042 ± 0.012 ^a^	0.059 ± 0.010 ^a^	0.047 ± 0.005 ^a^	0.061 ± 0.009 ^a^
Hexanal	6.380	0.416 ± 0.018 ^a^	0.775 ± 0.138 ^b^	0.811 ± 0.130 ^b^	0.704 ± 0.144 ^b^
Acids					
Dodecanoic acid	38.299	0.003 ± 0.000 ^a^	0.007 ± 0.001 ^a^	0.007 ± 0.001 ^a^	0.008 ± 0.003 ^a^
Acetic acid	16.654	7.369 ± 0.636 ^a^	15.059 ± 1.197 ^b^	17.012 ± 2.147 ^b^	19.261 ± 1.363 ^b^
n-Decanoic acid	34.616	0.037 ± 0.013 ^b^	0.148 ± 0.048 ^a^	0.114 ± 0.023 ^a^	0.172 ± 0.012 ^a^
Hexanoic acid	26.238	0.295 ± 0.025 ^a^	0.665 ± 0.098 ^b^	0.608 ± 0.050 ^b^	0.769 ± 0.105 ^b^
Isobutyric acid	19.807	3.097 ± 0.068 ^a^	1.943 ± 0.142 ^b^	2.517 ± 0.478 ^ab^	1.623 ± 0.187 ^b^
Butanoic acid	21.284	1.030 ± 0.078 ^b^	2.244 ± 0.162 ^a^	2.207 ± 0.088 ^a^	2.647 ± 0.162 ^a^
Pentanoic acid	24.135	0.007 ± 0.001 ^a^	0.011 ± 0.002 ^a^	0.009 ± 0.001 ^a^	0.011 ± 0.002 ^a^
Nonanoic acid	32.790	0.008 ± 0.002 ^a^	0.011 ± 0.001 ^a^	0.006 ± 0.001 ^a^	0.011 ± 0.002 ^a^
Propanoic acid	19.116	0.372 ± 0.030 ^b^	0.606 ± 0.014 ^a^	0.530 ± 0.038 ^ab^	0.521 ± 0.076 ^ab^
Octanoic acid	30.668	0.096 ± 0.017 ^a^	0.283 ± 0.093 ^a^	0.242 ± 0.050 ^a^	0.273 ± 0.035 ^b^
n-Hexadecanoic acid	44.738	0.008 ± 0.002 ^a^	0.011 ± 0.002 ^a^	0.009 ± 0.001 ^a^	0.007 ± 0.001 ^a^
Ketones					
2,3-Dehydro-1,8-cineole	9.529	0.020 ± 0.004 ^a^	0.033 ± 0.004 ^a^	0.030 ± 0.004 ^a^	0.035 ± 0.001 ^a^
2-Nonanone	15.281	0.029 ± 0.005 ^a^	0.018 ± 0.003 ^a^	0.024 ± 0.001 ^a^	0.048 ± 0.020 ^a^
Butanone	2.672	0.153 ± 0.030 ^b^	0.225 ± 0.020 ^ab^	0.345 ± 0.079 ^ab^	0.385 ± 0.043 ^a^
3-Hydroxy-2-butanone	12.261	4.193 ± 0.591 ^a^	2.528 ± 0.122 ^ab^	2.646 ± 0.566 ^ab^	1.885 ± 0.376 ^b^
Diacetyl	3.835	0.154 ± 0.037 ^a^	0.090 ± 0.010 ^a^	0.109 ± 0.025 ^a^	0.108 ± 0.026 ^a^
Hydroxyacetone	12.620	0.008 ± 0.001 ^a^	0.011 ± 0.002 ^a^	0.015 ± 0.005 ^a^	0.012 ± 0.001 ^a^
6-Methyl-5-hepten-2-one	13.833	0.002 ± 0.000 ^b^	0.004 ± 0.001 ^ab^	0.003 ± 0.000 ^ab^	0.006 ± 0.001 ^a^
Methyl isobutyl ketone	4.475	0.012 ± 0.002 ^a^	0.015 ± 0.003 ^a^	0.055 ± 0.014 ^a^	0.024 ± 0.001 ^ab^
Esters					
Benzeneacetic acid, ethyl ester	24.760	0.019 ± 0.003 ^a^	0.046 ± 0.011 ^a^	0.032 ± 0.006 ^a^	0.042 ± 0.009 ^a^
2,2,4-trimethyl-1,3-pentanediol diisobutyrate	26.839	0.019 ± 0.004 ^a^	0.044 ± 0.011 ^a^	0.038 ± 0.009 ^a^	0.041 ± 0.007 ^a^
Ethyl propionate	3.507	0.006 ± 0.002 ^a^	0.017 ± 0.002 ^b^	0.015 ± 0.002 ^b^	0.015 ± 0.002 ^b^
Ethyl isovalerate	6.107	1.723 ± 0.407 ^a^	1.865 ± 0.455 ^a^	1.934 ± 0.450 ^a^	1.638 ± 0.227 ^a^
Decanoic acid, ethyl ester	21.623	0.020 ± 0.003 ^a^	0.028 ± 0.003 ^a^	0.021 ± 0.002 ^a^	0.046 ± 0.015 ^a^
1,2-Benzenedicarboxylic acid, bis(2-methylpropyl) ester	38.781	0.006 ± 0.001 ^a^	0.018 ± 0.005 ^a^	0.017 ± 0.001 ^a^	0.019 ± 0.002 ^a^
1,6-Octadien-3-ol, 3,7-dimethyl-, formate	19.612	0.003 ± 0.001 ^a^	0.004 ± 0.001 ^a^	0.004 ± 0.000 ^a^	0.003 ± 0.001 ^a^
Dodecanoic acid, ethyl ester	26.180	0.004 ± 0.001 ^a^	0.004 ± 0.001 ^a^	0.005 ± 0.000 ^a^	0.005 ± 0.000 ^a^
Ethyl acetate	2.546	0.057 ± 0.015 ^b^	0.486 ± 0.147 ^a^	0.417 ± 0.092 ^a^	0.541 ± 0.054 ^a^
Nonanoic acid, ethyl ester	19.167	0.003 ± 0.001 ^a^	0.004 ± 0.001 ^a^	0.004 ± 0.001 ^a^	0.005 ± 0.002 ^a^
Octanoic acid, methyl ester	15.339	0.002 ± 0.000 ^a^	0.002 ± 0.000 ^a^	0.002 ± 0.000 ^a^	0.002 ± 0.000 ^a^
Heptyl acetate	8.305	0.003 ± 0.000 ^a^	0.006 ± 0.001 ^a^	0.005 ± 0.002 ^a^	0.005 ± 0.000 ^a^
Octanoic acid, 3-methylbutyl ester	12.817	0.005 ± 0.000 ^a^	0.005 ± 0.000 ^a^	0.003 ± 0.000 ^a^	0.006 ± 0.002 ^a^
Butanoic acid, 3-methyl-, 3-methylbutyl ester	12.745	0.015 ± 0.002 ^a^	0.004 ± 0.001 ^b^	0.003 ± 0.000 ^b^	0.003 ± 0.001 ^b^
1,5-Dimethyl-1-vinyl-4-hexenyl butyrate	24.435	0.006 ± 0.000 ^a^	0.011 ± 0.003 ^a^	0.008 ± 0.000 ^a^	0.007 ± 0.001 ^a^
Ethyl 2-methylpropanoate	3.680	0.052 ± 0.015 ^b^	0.144 ± 0.026 ^ab^	0.194 ± 0.058 ^a^	0.111 ± 0.008 ^a^
Propanoic acid, 2-methyl-, 3-methylbutyl ester	9.761	0.007 ± 0.003 ^a^	0.003 ± 0.001 ^a^	0.003 ± 0.000 ^a^	0.003 ± 0.000 ^a^
Isopentyl acetate	7.559	0.026 ± 0.007 ^a^	0.026 ± 0.003 ^a^	0.018 ± 0.002 ^a^	0.030 ± 0.012 ^a^
Ethyl hexanoate	10.930	0.027 ± 0.009 ^b^	0.049 ± 0.007 ^ab^	0.048 ± 0.006 ^ab^	0.064 ± 0.012 ^a^
Triethyl phosphate	22.242	0.002 ± 0.000 ^b^	0.005 ± 0.000 ^a^	0.004 ± 0.001 ^a^	0.005 ± 0.000 ^a^
Methyl isobutyrate	2.972	0.002 ± 0.000 ^a^	0.002 ± 0.000 ^a^	0.002 ± 0.000 ^a^	0.001 ± 0.000 ^a^
Ethyl octanoate	16.575	0.013 ± 0.002 ^b^	0.035 ± 0.006 ^a^	0.032 ± 0.005 ^ab^	0.041 ± 0.010 ^a^
Ethyl butanoate	5.258	0.047 ± 0.014 ^b^	0.146 ± 0.029 ^a^	0.130 ± 0.010 ^a^	0.171 ± 0.018 ^a^
Phenols					
Methyl eugenol	29.464	0.070 ± 0.002 ^b^	0.125 ± 0.007 ^a^	0.087 ± 0.006 ^ab^	0.121 ± 0.039 ^ab^
Eugenol	32.301	0.610 ± 0.272 ^b^	2.107 ± 0.246 ^a^	1.667 ± 0.089 ^a^	1.667 ± 0.157 ^a^
p-Cresol	30.719	0.035 ± 0.003 ^b^	0.068 ± 0.005 ^a^	0.055 ± 0.002 ^ab^	0.063 ± 0.013 ^a^

Data are expressed as relative peak area of each compound relative to the internal standard. Retention time (RT); *Staphylococcus carnosus* group (RS); *Staphylococcus xylosus* group (MS); Mixed strains group (HS); average ± standard error of mean; ^a–c^ same letters in the row indicate no significant difference (*p* > 0.05).

## Data Availability

The original contributions presented in the study are included in the article, further inquiries can be directed to the corresponding author.

## References

[1] Chen Q., Kong B., Han Q., Xia X., Xu L. (2017). The role of bacterial fermentation in lipolysis and lipid oxidation in Harbin dry sausages and its flavour development. LWT Food Sci. Technol..

[2] Yang Y., Sun Y., Pan D., Wang Y., Cao J. (2018). Effects of high pressure treatment on lipolysis-oxidation and volatiles of marinated pork meat in soy sauce. Meat Sci..

[3] Wang Y., Jiang Y., Cao J., Chen Y., Sun Y., Zen X., Pan D., Ou C., Gan N. (2016). Study on lipolysis-oxidation and volatile flavour compounds of dry-cured goose with different curing salt content during production. Food Chem..

[4] Lorenzo J.M., Gómez M., Purriños L., Fonseca S. (2016). Effect of commercial starter cultures on volatile compound profile and sensory characteristics of dry-cured foal sausage. J. Sci. Food Agric..

[5] Tatiyaborworntham N., Oz F., Richards M.P., Wu H. (2022). Paradoxical effects of lipolysis on the lipid oxidation in meat and meat products. Food Chem. X.

[6] Chen C., Fan X., Hu Y., Zhou C., Sun Y., Du L., Pan D. (2023). Effect of different salt substitutions on the decomposition of lipids and volatile flavor compounds in restructured duck ham. LWT Food Sci. Technol..

[7] Camargo V.P., Catanio N., de Marins A.R., Bergamasco R., Gomes R., Feihrmann A.C. (2021). The Physicochemical and sensory characteristics of Coppa with *Bifidobacterium animalis* ssp. Lactis (BB12) probiotic. Acta Scientiarum Technol..

[8] Flores M., Piornos J.A. (2021). Fermented meat sausages and the challenge of their plant-based alternatives: A comparative review on aroma-related aspects. Meat Sci..

[9] Li Z., Wang Y., Pan D., Geng F., Zhou C., Cao J. (2022). Insight into the relationship between microorganism communities and flavor quality of Chinese dry-cured boneless ham with different quality grades. Food Biosci..

[10] Toledano A.M., Jordano R., Medina L.M., López-Mendoza M.C. (2019). Behavior and effect of combined starter cultures on microbiological and physicochemical characteristics of dry-cured ham. J. Food Sci. Technol..

[11] Li Y., Cao Z., Yu Z., Zhu Y., Zhao K. (2023). Effect of inoculating mixed starter cultures of *Lactobacillus* and *Staphylococcus* on bacterial communities and volatile flavor in fermented sausages. Food Sci. Hum. Wellness.

[12] Mainar M.S., Stavropoulou D.A., Leroy F. (2017). Exploring the metabolic heterogeneity of coagulase-negative *staphylococci* to improve the quality and safety of fermented meats: A review. Int. J. Food Microbiol..

[13] Gao P., Wang W.X., Xia W.S., Xu Y.S., Jiang Q.X. (2016). Lipolysis and lipid oxidation caused by *Staphylococcus xylosus* and *Saccharomyces cerevisiae* isolated from Suan yu, a traditional Chinese low-salt fermented fish. Int. J. Food Sci. Technol..

[14] Lorenzo J.M., Gómez M., Fonseca S. (2014). Effect of commercial starter cultures on physicochemical characteristics, microbial counts and free fatty acid composition of dry-cured foal sausage. Food Control.

[15] Ferreira de Campos T.A., Rech de Marins A., Marques da Silva N., Matiucci M.A., Catarini Dos Santos I., Alcalde C.R., Rodrigues de Souza M.L., Gomes R.G., Feihrmann A.C. (2022). Effect of the addition of the probiotic *Bifidobacterium animalis* subsp. *Lactis* (BB-12) in free and microencapsulated form and the prebiotic inulin to synbiotic dry coppa. Food Res. Int..

[16] Motilva M.J., Toldrá F., Flores J. (1992). Assay of lipase and esterase activities in fresh pork meat and dry-cured ham. Z. Für Leb. Und Forsch..

[17] Gata J., Pinto M., Macias P. (1996). Lipoxygenase activity in pig muscle: Purification and partial characterization. J. Agric. Food Chem..

[18] Martín A., Colín B., Aranda E., Benito M.J., Córdoba M.G. (2007). Characterization of *Micrococcaceae* isolated from Iberian dry-cured sausages. Meat Sci..

[19] Cruxen C., Funck G.D., Haubert L., Dannenberg G.D.S., Marques J.L., Chaves F.C., da Silva W.P., Fiorentini Â.M. (2019). Selection of native bacterial starter culture in the production of fermented meat sausages: Application potential, safety aspects, and emerging technologies. Food Res. Int..

[20] Wang J., Lu S., Wang Q., Guo X., He J. (2020). Effects of starter cultures on lipid oxidation and accumulation of biogenic amines in traditional Chinese smoked horsemeat sausage. J. Food Process. Preserv..

[21] Zhang J., Pan D., Zhou G., Wang Y., Dang Y., He J., Li G., Cao J. (2019). The Changes of the Volatile Compounds Derived from Lipid Oxidation of Boneless Dry-Cured Hams During Processing. Eur. J. Lipid Sci. Technol..

[22] Zhou G.H., Zhao G.M. (2007). Biochemical changes during processing of traditional Jinhua ham. Meat Sci..

[23] Liu Q., Lei M., Lin J., Zhao W., Zeng X., Bai W. (2023). The roles of lipoxygenases and autoxidation during mackerel (*Scomberomorus niphonius*) dry-cured processing. Food Res. Int..

[24] Xiao Y., Liu Y., Chen C., Xie T., Li P. (2020). Effect of *Lactobacillus plantarum* and *Staphylococcus xylosus* on flavour development and bacterial communities in Chinese dry fermented sausages. Food Res. Int..

[25] Wang D., Zhao L., Su R., Jin Y. (2019). Effects of different starter culture combinations on microbial counts and physico-chemical properties in dry fermented mutton sausages. Food Sci. Nutr..

[26] Wang H., Su W., Mu Y., Zhao C. (2021). Correlation Between Microbial Diversity and Volatile Flavor Compounds of Suan zuo rou, a Fermented Meat Product from Guizhou, China. Front. Microbiol..

[27] Hu Y., Wang H., Kong B., Wang Y., Chen Q. (2021). The succession and correlation of the bacterial community and flavour characteristics of Harbin dry sausages during fermentation. LWT Food Sci. Technol..

[28] Ma Y., Gao Y., Xu Y., Zhou H., Zhou K., Li C., Xu B. (2023). Microbiota dynamics and volatile metabolite generation during sausage fermentation. Food Chem..

[29] Marco A., Navarro J.L., Flores M. (2004). Volatile compounds of dry-fermented sausages as affected by solid-phase microextraction (SPME). Food Chem..

[30] Wen R., Yin X., Hu Y., Chen Q., Kong B. (2022). Technological properties and flavour formation potential of yeast strains isolated from traditional dry fermented sausages in Northeast China. LWT Food Sci. Technol..

[31] Curioni P.M.G., Bosset J.O. (2002). Key odorants in various cheese types as determined by gas chromatography-olfactometry. Int. Dairy J..

[32] Flores M., Corral S., Cano-García L., Salvador A., Belloch C. (2015). Yeast strains as potential aroma enhancers in dry fermented sausages. Int. J. Food Microbiol..

[33] García-González D.L., Aparicio R., Aparicio-Ruiz R. (2013). Volatile and amino acid profiling of dry cured hams from different swine breeds and processing methods. Molecules.

